# Heart Failure with Preserved Ejection Fraction – Concept, Pathophysiology, Diagnosis and Challenges for Treatment

**DOI:** 10.3889/oamjms.2015.087

**Published:** 2015-07-28

**Authors:** Lidija Veterovska Miljkovik, Vera Spiroska

**Affiliations:** 1 *Institute for Gerontology “13. November”, Skopje, Republic of Macedonia*; 2 *University Clinic of Cardiology, Faculty of Medicine, Ss Cyril and Methodius University of Skopje, Skopje, Republic of Macedonia*

**Keywords:** hearth failure, diastole, preserved ejection fraction, echocardiography, old people

## Abstract

Heart failure (HF) with preserved left ventricular (LV) ejection fraction (HFpEF) occurs in 40 to 60% of the patients with HF, with a prognosis which is similar to HF with reduced ejection fraction (HFrEF). HFpEF pathophysiology is different from that of HFrEF, and has been characterized with diastolic dysfunction. Diastolic dysfunction has been defined with elevated left ventricular stiffness, prolonged iso-volumetric LV relaxation, slow LV filing and elevated LV end-diastolic pressure. Arterial hypertension occurs in majority cases with HFpEF worldwide. Patients are mostly older and obese. Diabetes mellitus and atrial fibrillation appear proportionally in a high frequency of patients with HFpEF. The HFpEF diagnosis is based on existence of symptoms and signs of heart failure, normal or approximately normal ejection and diagnosing of LV diastolic dysfunction by means of heart catheterization or Doppler echocardiography and/or elevated concentration of plasma natriuretic peptide. The present recommendations for HFpEF treatment include blood pressure control, heart chamber frequency control when atrial fibrillation exists, in some situations even coronary revascularization and an attempt for sinus rhythm reestablishment. Up to now, it is considered that no medication or a group of medications improve the survival of HFpEF patients. Due to these causes and the bad prognosis of the disorder, rigorous control is recommended of the previously mentioned precipitating factors for this disorder. This paper presents a universal review of the most important parameters which determine this disorder.

## Introduction

Beside contemporary treatment modalities, the heart failure (HF) is still a progressive disorder with a high morbidity and mortality rate [1]. Because of a great number of older people worldwide, it is expected that the incidence and the prevalence of the heart failure (HF) will increase rapidly in the next decade [2]. Beside the improvement of medical treatment, the mortality rate from this disorder has been still unacceptably high and becomes a leading cause for death in older people [1]. A great number of studies proved the most frequent risk-factors, being associated with the appearance of HF, such as advanced age, hypertension and ischemic heart disease [2].

In about 50% of the patients having the symptoms and signs for heart failure, normal or approximately normal values of ejection fraction, when a separate clinical entity was isolated, called a heart failure with preserved ejection fraction (HFpEF). Numerous studies point the fact that it is a disorder with a complex pathophysiology, on which progress and prognosis impact more cardiovascular disturbances [1]. It is expected that in the next decade HFpEF will become a dominant cause for heart failure worldwide, and due to that it becomes a provocative and important healthy problem for which, still, no treatment has been established, which will improve the prognosis of this disorder [1].

Up to now, it is considered that no medication or a group of medications improve the survival of HFpEF patients. Due to these causes and the bad prognosis of the disorder, rigorous control is recommended of the previously mentioned precipitating factors for this disorder. This paper presents a universal review of the most important parameters which determine this disorder.

## Material and Methods

Investigations in medical electronic data basis (Pub Med, Google Scholar, Plos, and Elsevier) showed a great number of articles, especially in the last decade, which analyzed these subjects. In this review, 28 articles are cited, all published in the indexed world journals.

Years backwards, the treatment of the heart failure was directed towards treatment of systolic dysfunction [3]. Historically viewed, a systolic dysfunction with EF < 45% was considered for heart failure. In line with Roelandt, the first association between myocardial relaxation and ventricular function was described in 1923 by Yendel Handerson, who presented data that myocardial relaxation was equally important as well as the contraction [4]. Gaasch defined the term “systolic dysfunction” in 1994 as “the inability of the heart to adapted to the blood volume during diastole and the ventricular filing was delayed and incomplete, the atrial pressure was growing, causing pulmonary or systemic congestion”. Ten years later, in 2004, the same author “redefined” this entity adding “diastolic dysfunction could occur when the ejection fraction was normal or disturbed”. In 1980, medical publicity started to recognize the symptoms and signs for heart failure in patients with normal ejection fraction [3]. Contrary to HFrEF, the individuals with HFpEF were generally older, more frequently women, and had increased incidence for developing hypertension, diabetes, coronary arterial disease, obesity and atrial fibrillation [5].

Asymptomatic patients with hypertensive left ventricular hypertrophy that, by echocardiography, show normal ejection fraction and disturbed left ventricular filing, could be said to have diastolic dysfunction [6, 7]. If these patients develop intolerance to effort, dyspnea, with venous or pulmonary congestion, it is considered to have “diastolic heart failure” [8].

Prevalence of diastolic heart failure is higher in people older than 75 years. The mortality rate in patients with diastolic heart failure ranges between 5 and 8%, compared to the patients with systolic heart failure, in whom it ranges between 10 and 15%. Morbidity, including the frequency of hospitalizations, has been approximately equal in these two groups of patients [8].

**Figure 1:**
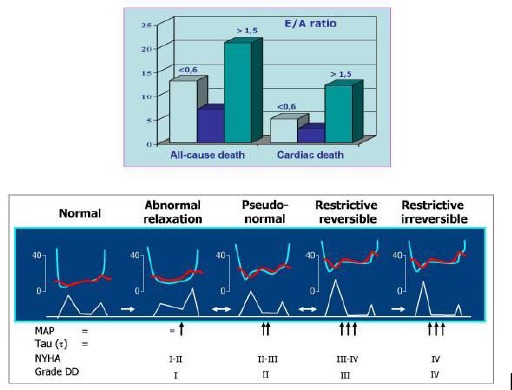
*Results from the investigation of the cardiac mortality depending the grade of diastolic dysfunction. NYHA-functional class for heart failure; MAP medium atrial pressure; DD diastolic dysfunction [2, 7]*.

In a greater cohort study – PREVEND - an analysis has been made for the causes of HFpEF occurring within the period of 11 years, the investigation showed that the advanced age, urinary albumin excretion, cystatin C and a history of atrial fibrillation were strongly indicated by occurrence of HFpEF. In the past two decades, the percent of the patients with preserved ejection fraction increased from 38% to 54% of all the cases with hearth failure and this percent was considered to increase progressively with aging of the population and with the increase of the prevalence of patients with hypertension, obesity and diabetes [2, 9].

## Pathophysiology

Diastolic dysfunction, defined by increased left ventricular stiffness, prolonged the iso-volumetric LV relaxation; slow LV filing and elevated LV end-diastolic pressure lie in the basis of HFpEF [6, 10]. LV diastole consists of iso-volumetric relaxation and ventricular filing. LV relaxation is an active process occurring due to energetically dependent intracellular calcium input in sarco-plasmatic reticulum, which concentration increases during systole [11]. LC is disturbed by diseases which disturb the energetic metabolism of calcium re-uptake such as myocardial ischemia and myocardial hypertrophy [6]. Aging, on its own, is directly connected with cardiac contractile dysfunction, which is manifested with prolonged time duration of the left chamber relaxation and stress intolerance [9]. It is proved, in trials, that aging, on its own, leads to diastolic dysfunction, oxidative stress and protein modification [9]. Left ventricular filing as a dynamic phase of diastole has been disturbed most frequently due to myocardial fibrosis or hypertrophy [11].

Myocardial fibrosis has been caused by humoral factors such as various types of cytokines, “growth factors” and hormones. In hypertensive HFpEF, the oxidative stress increases the angiotensin II secretion from the blood vessels walls, due to elevated blood pressure. This results in fibroblast activation and increased protein-1 production of the transforming “growth factor” from macrophages and activation of monocyte haemotaxic protein-1. This results in perivascular inflammation, which is considered as a cause for reactive myocardial fibrosis [11].

**Figure 2:**
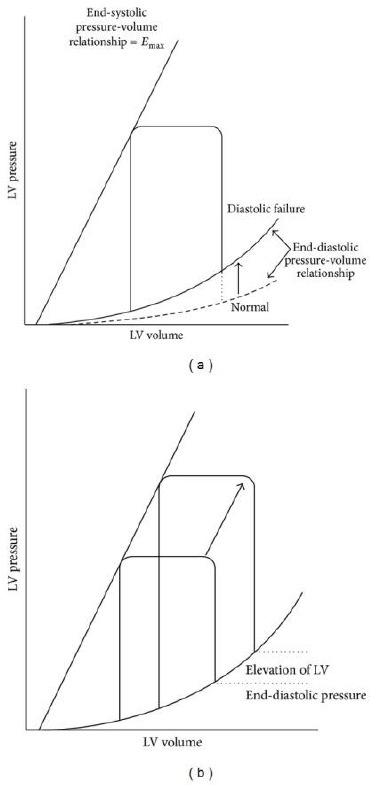
*End-systolic relation pressure-volume is equal in normal heart and diastolic heart failure, but the end-diastolic relation pressure-volume is different [11]*.

Ventricular filing in diastole has been defined by physical characteristics of the heart cavities, which establishes the relation between pressure and volume. These physical characteristics depend on ventricular compliance, which means that the relation among some volume variations, appropriately correspond with the pressure changes. If, small volume variations, cause significant changes in diastolic pressure for longer period, there is a tendency for the patients to develop venous congestion and diastolic heart failure [12].

When the left ventricular function has been disturbed, the heart “output” is decreased concerning the incomplete filing of the LC in diastole, and as a compensatory mechanism elevates the LC end-diastolic pressure, consequently the left atrial pressure also elevates and as a final result a direct pulmonary congestion.

In the recent years, the new studies changed the concept, which traditionally was associated with development of heart failure with preserved systolic function [13]. These studies showed that the heart failure with preserved systolic function was a separate entity, which pathophysiology was complex and not to the end elucidated, and which needed different clinical approach from that which was applied for heart failure with impaired ejection fraction. Diastolic dysfunction has a central role in pathophysiology of heart failure with preserved ejection fraction, although it, on its own, could not be a cause to develop a clinical picture of heart failure. In that sense, it is important to emphasize that there is a great number of patients who have diastolic dysfunction, are asymptomatic and do not have signs for heart failure, even more that the prevalence of diastolic dysfunction surpasses 25% of the general population [13]. Among the “extra hearth” disorders which are pointed in pathophysiology of the heart failure with preserved systolic function are: endothelial dysfunction, changes of the blood vessel walls and reduced vasodilatation reserve [6].

## Diagnosis of heart failure with preserved ejection fraction (HFpEF)

Recommendations for a diagnosis of heart failure with preserved ejection fraction (HFpEF) are proved by the European Society for Cardiology (ESC) in 2012 and by the American Academy of Cardiology (ACC/AHA) in 2013. The diagnosis of HFpEF is based on existence of symptoms and signs for heart failure, normal or approximately normal ejection fraction (EF > 45-50%) and diagnosing of LV diastolic dysfunction by means of heart catheterization or Doppler echocardiography and/or elevated natriuretic peptide (BNP > 200 pg/ml) concentration As supplement were added the known criteria of the New York Heart Association (NYHA), which was more functional classification.

Diagnosing of LV diastolic dysfunction has been made most frequently with Doppler echocardiography and/or elevated natriuretic peptide concentration. Cardiac magnetic resonance imaging (CMRI) is a new modality for diagnosing of diastolic dysfunction [14].

**Figure 3:**
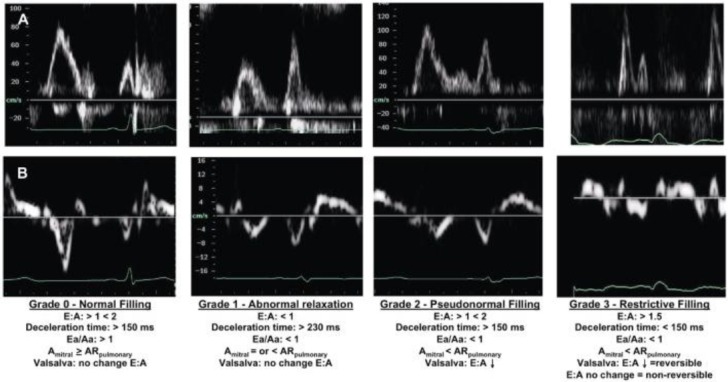
*Grades of diastolic dysfunction by means of pulsed wave mitral Doppler and tissue Doppler mitral annular measurement [17]*.

Echocardiography is the most important diagnostic method in patients with heart failure, as to the fact that the physical exam, the electrocardiogram and the X-ray do not give sufficient information, to distinguish the systolic from diastolic heart failure [12].

Establishment of diastolic function of the left chamber is an integral routine element of the echocardiographic evaluation of each patient.

Four grades of diastolic dysfunction are determined by echocardiography: First grade of diastolic dysfunction or disturbed relaxation (E/A < 0.8, DT > 200 ms, IVRT > 100 ms, S > D, e’ < 8 cm/s, Ar-A < 30m sec). Second grade of diastolic dysfunction or pseudo-normalization (E/A 0.8-1.5, DT-normal, S/D < 1, Ar-A > 30cm/s, E/e is increasing to 9-12, can be bigger). Third to fourth grade of diastolic dysfunction or restrictive ventricular filing EA >2, IVRI <60 ms, DT <160 ms reduced or none, septal E/e’ >15). In this grade, LC relaxation has been seriously disturbed while significantly increased the ventricular pressure.

The flow through mitral valve in diastole depends on transvalvular pressure gradient [4]. In normal conditions, this gradient is maximal immediately after the mitral valve opening, when corresponds with E-wave. Then, a period of reduced transvalvular gradient follows, which denotes a null flow (diastasis). Atrial contraction, which follows after that, causes a new gradient and flow, which corresponds to A-wave. The E/A ratio is considered to be a primary indicator for diastole. If disturbance of ventricular relaxation occurs, it comes to reduction of transvalvular gradient at the beginning of diastole, which consequently leads to reduction of the E-wave. Due to this, the atrial discharge disturbs, as a result to that the atrial contraction should be stronger, which is manifested with elevation of the A-wave. E/A ratio changes which is characteristically for the first grade of diastolic dysfunction. If the left atrial pressure is elevated for longer time, it causes symptoms of intolerance for physical effort (second grade of diastolic dysfunction). In patients in whom the LV diastolic pressure is so elevated, in order to restrict the transvalvular flow during the atrial contraction and constantly elevated left atrial pressure, it is called the third or restrictive type of diastolic dysfunction which is manifested with harder intolerance for physical effort [4, 6].

The analysis of the flow through pulmonary veins by means of pulsed Doppler, is an indirect indicator for atrial pressure, which, in absence of mitral valvular change, corresponds to LV diastolic pressure [4]. Pulsed Doppler of pulmonary flow measures the “peak systolic” (S), “peak anterograde diastolic” (D) and “peak atrial reversal” (Ar) velocities in apical four-cavity space, which is a good indicator of diastolic dysfunction [17, 18].

Other important indicators of diastolic dysfunction are tissue Doppler mitral annular measurement as well as the left atrial (LA) volume measurement and LA functional discharge index [12]. The tissue Doppler (TDI) measures the mitral annular velocities including the systolic, early diastolic (e’) and late diastolic (a) annular velocity. Patients with (e’) septal < 8 cm/s have disturbed left ventricular relaxation. The Association of Echocardiography in scope of the European Society of Cardiology regulated that the ratio E/e’ < 8 has been characterized with normal left ventricular filing, while the E/e’ > 15 denotes elevated left ventricular pressure during filing [12, 18].

**Figure 4:**
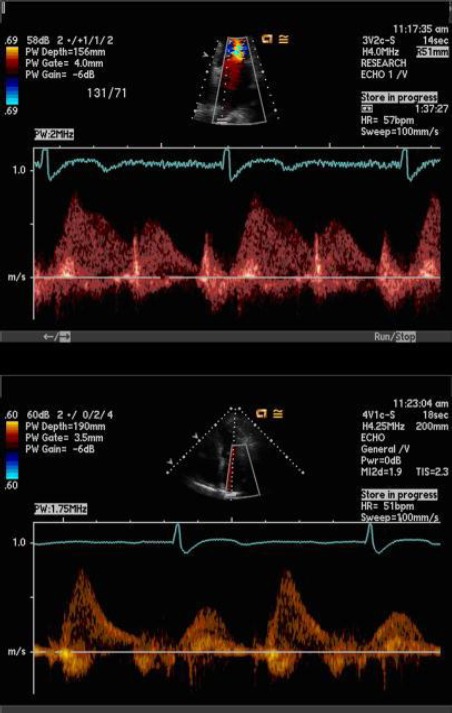
*Dopler of pulmonary vein [17]*.

Total atrial, passive and active fractions of discharge are reduced in patients with HFpEF compared to hypertensive patients with normal diastole function [12]. Many studies showed that the atrial volume and function were similar in patients with left ventricular hypertrophy and the control group, and were disturbed for 68% in patients with HFpEF [10]. Some studies indicated that LAFI /left-atrial functional index/, as well as B-natriuretic peptide concentration, were prognostic indicators within the survival time of the patients with HFpEF [12].

Atrial function maybe is a key compensatory mechanism, which determines the course and prognosis of the HFpEF patients [21]. Left atrium dysfunction, measured through the left atrial functional index, is associated with increased number of hospitalizations [12, 22]. It was also showed, in studies, that compared to the B-natriuretic peptide and to Doppler verified diastolic heart failure, the left atrial functional index (LFI) has been a better predictive factor for development of symptomatic, stable, chronic heart failure in older population [9].

In the past years, several studies pointed the significant connection of some biomarkers by appearance of the heart failure subtypes [11]. In line with them, the plasma B-natriuretic peptide concentration in HFpEF was lower than in the patients with HFrEF [11]. Highly sensitive troponin T was significantly associated with development of HFrEF. On the other hand, the growing factor (GDF 15), cystatin C, and the urinary albumin excretion were significantly associated with development of HFpE [7].

## Treatment modalities

Beside the fact that HFpEF is a frequent form of heart failure, and its frequency is expected to be in significant rise in the next decades, regarding the greater number of older population, as well as the number of the accompanying cardiovascular disorders, such as hypertension and diabetes, its pathophysiology is no completely defined, which is the main reason for lacking an approved therapeutic protocol.

Angiotensin-converting enzyme inhibitors (ACEI), angiotensin receptor blockers (ARB), beta-blockers and statins are used in the HFpEF treatment. But even beside that, none of them is authorized in reduction of mortality rate and the HFpEF patients’ survival.

Hypertension treatment is one of the most important factors in treatment of diastolic dysfunction [20]. Effective decrease of blood pressure reduces the left atrial and left end-diastole pressure, improves the LV relaxation and filling. Also, reduction of blood pressure reduced the LV hypertrophy and the risk for progression of heart failure is decreasing [22]. ACEI Perindopril was tested in a trial of 850 patients with HFpEF older than 70 years within the period of 26 months. This trial did not show difference in mortality and/or the number of hospitalizations from heart failure in the examinees compared to the control group. CHARM study, which investigated the ARB Candesartan impact in 3023 patients (EF > 40%), but did not show reduction of cardiovascular death in HFpEF patients, only reduction of number of hospitalizations of the treated group compared to the control one. In the PRESERVE study, the group of patients who were treated by Irbesartan was investigated, and then compared to the control one, the mortality rate and hospitalizations did not differ between them. Having in mind the HFpEF pathophysiology, beta blockers could have an effect, such as: 1) to reduce the heart frequency, by what the diastolic duration continues and corresponding to that the time of ventricular filling; 2) to reduce the myocardial oxygen demand 3); to reduce the blood pressure and bring to regression the LC hypertrophy [5]. In addition, there are not sufficient trials with these medications, which will prove, with certainty, their benefits on mortality reduction of the HFpEF patients.

Aldosterone is found to play a role in occurrence of cardiac fibrosis [16]. It is found that the aldosterone level showed a rise with advance of the age [16]. Higher aldosterone concentrations were found in patients with diastolic heart failure, compared to the control group, but not so high, as in the patients with systolic heart failure. Also, aldosterone was found to increase the LV mass in hypertensive patients independently than the plasma renin activity [16]. Accordingly, blockade of aldosterone could improve the cardiac fibrosis and LV performances [16]. Efficacy and safety of the long term aldosterone receptor blockade in HFpEF patients was investigated in Aldo-DHF randomized, multicentric, prospective study. By this treatment, the study showed that the left ventricular diastolic function and the left ventricular re-modeling improved, but, however, the intolerance for physical effort, the patients’ symptoms and the patients’ quality of life did not. In a study of 245 patients having heart failure with ejection fraction >40%, treated with Carvedilol in standard doses within a period of 3.2 years, compared to the control group which was not treated with Carvedilol, the prognosis did not improve in HFpEF patients [24].

In Randomized Aldactone Evaluation Study, correlation was found between the clinical benefit from the spironolactone use on serum markers for cardiac fibrosis, but other studies were lacking, which, with certainty, would determine the place of aldosterone inhibitors in the treatment of HFpEF patients [16]. This indicated that the data-base of the total of 53878 patients examined in 30 published studies (18 randomized controlled and 12 observed studies), showed that combined therapy could improve the symptoms of the HFpEF patients, but not the mortality rate [10, 25]. New modalities of HFpEF treatment in future could depend on profound knowledge for pathophysiology of diastolic heart failure.

Some new medications promise to be used in the treatment of HFpEF in future. Phospodiesterase 5 inhibitors are considered to reduce LV and arterial stiffness, to improve the endothelial function and reduce the pulmonary vascular resistance /RELAX-study/ [10, 26]. Alagebrium chloride is a new agent which, in experimental research in animals, was found to improve the ventricular and vascular compliance, to reduce the blood pressure and vascular stiffness [20, 27].

## Prognosis and discussion

Experimental studies being conducted worldwide in the past decade ruined the previous concept that the prognosis of the heart failure with preserved ejection fraction was better than that of the patients with reduced ejection fraction. The facts worry that the survival of the patients with heart failure with preserved ejection fraction was not better in the last decades, opposite to longer survival of the patients with systolic heart failure.

In patients with heart failure, diastolic dysfunction is a bigger prognostic indicator for mortality than the ejection fraction [8, 27].

There are controversies in literature concerning the terminology, such as “Diastolic heart failure” or Heart failure with preserved ejection fraction”. The authors who preferred the former term probably did not take into account the fact that some systolic disturbances almost always were present in the patients with diastolic heart failure which were frequently proved by echocardiography, which spoke that this was a complex phenomenon that should be viewed as one entity [11].

Diastolic and systolic heart failure should not be considered as completely separate entities, diastolic function is disturbed even in systolic heart failure and is considered that it stresses even more the intolerance to physical effort and determines the prognosis.

**Figure 5:**
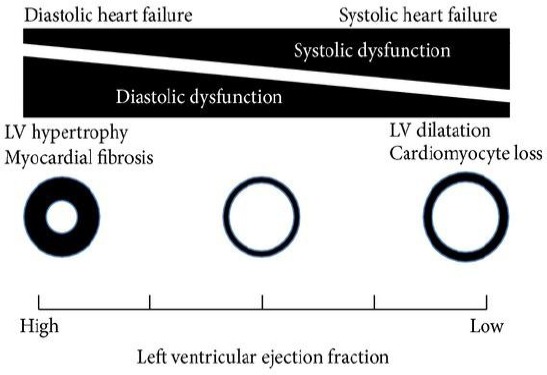
*Systolic and diastolic heart failure-two phenotypes with different pathophysiology that are together linked [11]*.

On the other hand, systolic contractile disturbances measured by tissue Doppler are also detected in diastolic heart failure. Accordingly, it comes out that the heart failure is the only entity with two phenotypes, which complement to each other [11].
